# Malignancy in the blind painful eye – report of two cases and literature review

**DOI:** 10.1186/1746-1596-1-45

**Published:** 2006-11-21

**Authors:** Patrícia Rusa Pereira, Alexandre Nakao Odashiro, João Pessoa Souza Filho, Vinicius S Saraiva, David Gerardo Camoriano, Miguel N Burnier

**Affiliations:** 1 Department of Ophthalmology, Federal University of São Paulo, São Paulo, Brazil; 2 Henry C. Witelson Ocular Pathology Laboratory, Department of Ophthalmology McGill University, Montreal, Canadá; 3 LAC, Pathology and Cytopathology Laboratory, Campo Grande, MS, Brazil; 4 Universidade para o Desenvolvimento do Estado e Região do Pantanal, UNIDERP, Campo Grande, MS, Brazil

## Abstract

**Background:**

Few cases of malignant tumors arising in a blind painful eye have previously been described. We described two cases of a blind painful eye containing an unsuspected tumor, which were enucleated to relieve the pain.

**Case presentations:**

**Case 1**: A 57 year-old Caucasian man presented with recurrent orbital cellulitis and endophthalmitis in the left eye (OS). The OS was blind and painful and an enucleation was performed showing a uveal melanoma by histopathological exam. **Case 2**: A 54 year-old Caucasian man with previous history of a rhegmatogenous retinal detachment in his left eye presented a blind painful eye. Enucleation was performed revealing a well-differentiated B-cell lymphoma of uveal tract with extra ocular extension.

**Conclusion:**

In the management of a blind painful eye, it is extremely important to rule out an intraocular malignancy particularly in those patients who have not been followed by an ophthalmologist.

## Background

A blind eye may be associated with pain, which is a challenge for the ophthalmologist. The most common conditions leading to the development of a blind painful eye (BPE) are trauma, miscellaneous retinal disorders and retinal detachment, and the majority of these eyes are enucleated to relieve the pain.[1]

Few cases of malignant tumors arising from BPE have previously been described. [2-7] From literature review, the frequency of unsuspected intraocular tumors in blind painful eyes has declined over the past twenty years mainly due to ocular ultrasound (US) examination. We described two cases of BPE containing unsuspected tumor, which were enucleated to relieve the pain.

## Case presentation

**Figure 1:**
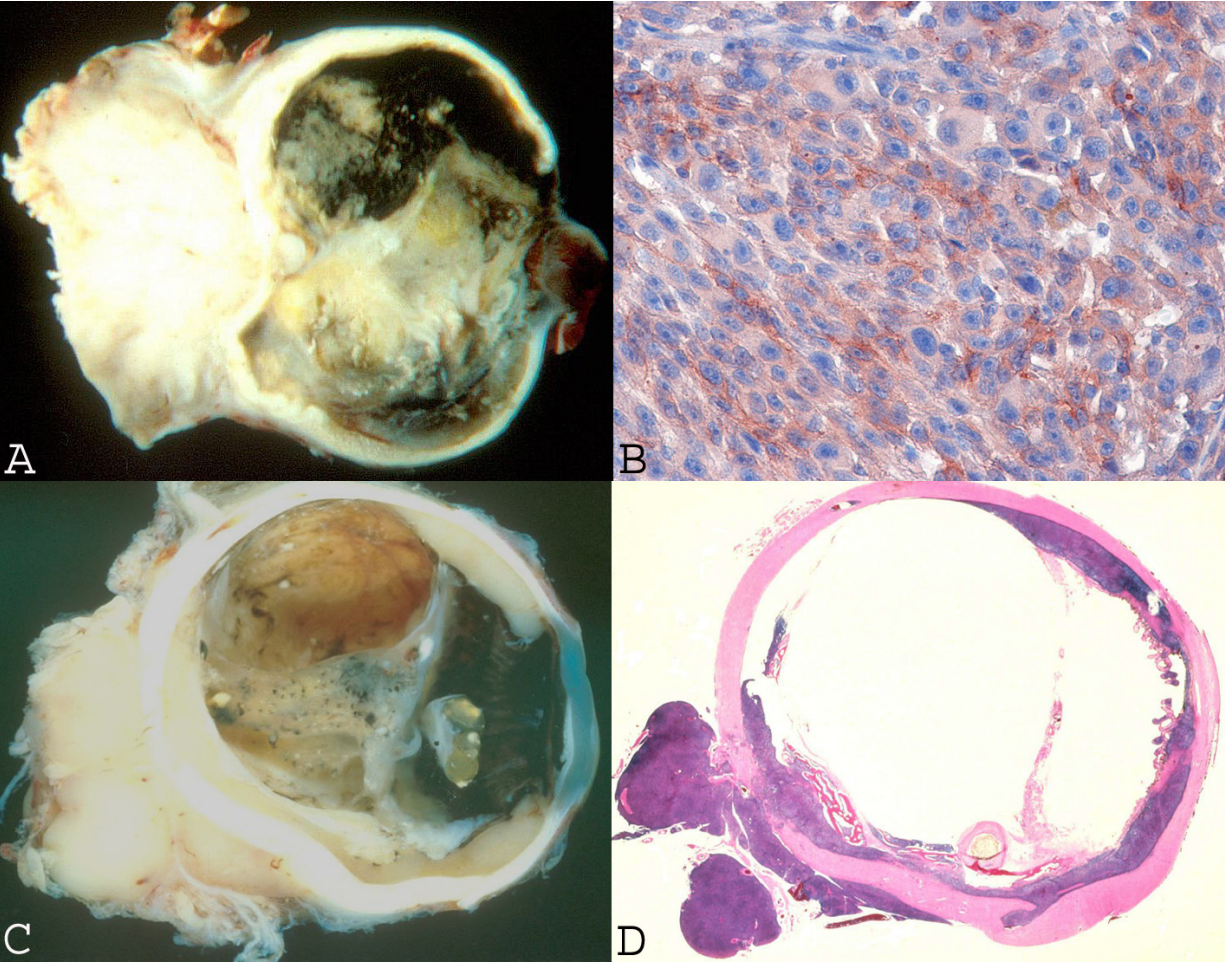
A: Gross examination discloses a pupil-optic nerve section presenting a pigmented mass on the choroid with extra-ocular extension. B: Photomicrograph of immunohistochemistry stained with HMB-45 showing strong reaction in almost all cells. The tumor is composed by epithelioid cells with large nuclei and conspicuous nucleoli. (Original magnification 200×). C: Gross examination of pupil-optic nerve section showing a whitish mass committing all uveal tract with extra-ocular extension. D: Haematoxylin and Eosin (H&E) preparation showing tumor in the uveal tract with extra-ocular extension.

**Case 1**: A 57 year-old Caucasian man had a previous history of cataract surgery, left eye (OS), in 1984. In 2000, the patient presented with recurrent orbital cellulitis and endophthalmitis OS. The OS was blind and painful and an enucleation was performed (Figure 1A). Histopathology revealed a malignant uveal melanoma, epithelioid cell type (Figure 1B), invading the sclera and orbital tissues. No signs of metastatic disease were detected after four years of follow-up.

**Case 2**: A 54 year-old Caucasian man suffering from Steinert's syndrome had a blind, atrophic OS since 1980. Past medical history includes systemic hypertension and several ophthalmic procedures OS, including a cataract surgery (1971) and a rhegmatogenous retinal detachment (1978). In 1992, the patient presented with pain in the OS and an uneventful enucleation was performed (Figure 1C). Histopathologic examination disclosed a monotonous and diffuse proliferation of small lymphocytes in the uveal tract with extra ocular extension. Immunohistochemical study was strongly positive for CD20 (B lymphocytes) and negative for CD45RO (T lymphocytes), consistent with a well-differentiated B-cell lymphoma (Figure 1D). No signs of systemic involvement were detected. The patient was lost to follow-up six years after surgery.

Severe pain may develop in blind eyes for various reasons including surgical and non-surgical trauma, and retinal detachment. Management of a blind painful eye represents a challenge for the ophthalmologist and is limited to topical medications, retrobulbar alcohol injection, evisceration or enucleation.[1]

Relief of ocular pain is the most common reason that enucleation is performed in BPE.[1] In the past, ocular melanomas arising in a BPE could account for more than 10% of all diagnosed uveal melanomas.[8] Volcker and Naumann[9] in 1976 described 36 cases of unsuspected ocular melanoma that were diagnosed after enucleation. The clinical diagnoses in those cases were secondary glaucoma (30), retinal detachment (2), iritis (2), and end/panophthalmitis (2). Review of the literature today shows that the clinical suspicion rate of an intraocular malignancy in BPE is not well established. Previous studies of enucleated globes do not correlate blindness with unsuspected intraocular tumors.[10]

In this particular report, the unsuspected melanoma was present in a blind painful eye of a mentally handicapped patient. Intraocular lymphomas of the uveal tract have been discovered in functional eyes with symptoms of retinal detachment and increased intraocular pressure.

Intraocular tumors arising in blind painful eyes are probably under diagnosed and underreported. Several uveal melanomas, [4,5,7] two adenocarcinomas of the retinal pigment epithelium [2,3] and an unspecified sarcoma [6] have been described (Table 1). In two of those cases, enucleation was performed to relieve the pain and an early stage malignant tumor was found,[2,7] leading to a good prognosis. However, in cases with advanced disease and extra-ocular involvement, an enucleation was performed due to a high index of suspicion of an intraocular malignancy.[4-6] In those cases the prognosis was poor.

**Table 1:** Malignant tumor in previous blind eyes

**Authors**	**Patient**	**Eye**	**Signs/Symptoms**	**Suspicious malignancy**	**Treatment**	**Pathologic diagnosis**	**Follow-up**
**Ten Thije^6^**	66-year-old, man	RE	Exophthalmos	Yes	Exenteration	Large-cell sarcoma	Death few months after diagnostic
**Sarma *et al*^5^**	62-year-old, man	LE	Progressive proptosis, eye pain, left orbit mass	Yes	Exenteration	Extrascleral Uveal melanoma	No follow-up reported
**Nelson & Kincaid^4^**	70-year-old, man	LE	dark inferonasal and superiorly conjunctival mass, mass in the anterior chamber	Yes	Exenteration	Extrascleral Uveal melanoma (ciliary body)	Pulmonary and bone metastasis, death 1 year after diagnostic
**Nelson & Kincaid^4^**	79-year-old, man	RE	eye pain, black exophytic subconjunctival mass	Yes	Exenteration	Extrascleral Uveal melanoma	Liver metastasis 8 months later, death 14 months after diagnostic
**Loeffler *et al*^3^**	66-year-old, man	RE	eye pain	No	Enucleation	Malignant tumor of the retinal pigment epithelium	No death or metastasis 1 year after enucleation
**Edelstein *et al*^2^**	79-year-old, woman	RE	eye pain, exophthalmos	No	Enucleation	Presumed Adenocarcinoma of the retinal pigment epithelium with staphyloma	No follow-up reported
**Tripathi *et al*^7^**	45-year-old	RE	Eye pain	No	Enucleation	Uveal melanoma	No follow-up reported
**Pereira *et al***	57-year-old, man	LE	eye pain, endophthalmitis, orbital cellulites	No	Enucleation	Extrascleral Uveal melanoma	No signs of metastatic disease after four years of follow-up
**Pereira *et al***	54-year-old, man	LE	eye pain	No	Enucleation	Extranodal lymphoma of uveal tract with extra ocular extension	Lost to follow-up after six years

Several authors emphasized the importance of ultrasonographic studies to diagnose intraocular tumors in blind painful eyes.[5,11]

## Conclusion

In the management of a blind painful eye, it is extremely important to rule out an intraocular malignancy particularly in those patients who have not been followed by an ophthalmologist. In these cases, it is the duty of the attending physician to emphasize to the patient the importance of regular examination of the blind eye because, like in all malignancies, advanced disease leads to a worse prognosis.[4] The present report also emphasizes the importance of subjecting enucleated globes to a histopathological examination, since an unsuspected intraocular malignancy may be hidden in a blind painful eye.

## Competing interests

The author(s) declare that they have no competing interests.

## Authors' contributions

PRP wrote the manuscript

ANO, JPSP and VS revised the histopathology of the cases and the manuscript

DGC prepared the pictures and revised the manuscript

MNB revised the entire manuscript
